# Spatial-temporal dynamics of neotropical velvet ant (Hymenoptera: Mutillidae) communities along a forest-savanna gradient

**DOI:** 10.1371/journal.pone.0187142

**Published:** 2017-10-27

**Authors:** Júlio Miguel Alvarenga, Cecília Rodrigues Vieira, Leandro Braga Godinho, Pedro Henrique Campelo, James Purser Pitts, Guarino Rinaldi Colli

**Affiliations:** 1 Programa de Pós-Graduação em Ecologia e Conservação, Departamento de Biologia, Universidade do Estado de Mato Grosso, Nova Xavantina, Mato Grosso, Brazil; 2 Department of Biology, Utah State University, Logan, Utah, United States of America; 3 Programa de Pós-Graduação em Biodiversidade, Ecologia e Conservação, Universidade Federal do Tocantins, Porto Nacional, Tocantins, Brazil; 4 Departamento de Zoologia, Universidade de Brasília, Brasília, Distrito Federal, Brazil; Chinese Academy of Agricultural Sciences Institute of Plant Protection, CHINA

## Abstract

Understanding how and why biological communities are organized over space and time is a major challenge and can aid biodiversity conservation in times of global changes. Herein, spatial-temporal variation in the structure of velvet ant communities was examined along a forest-savanna gradient in the Brazilian Cerrado to assess the roles of environmental filters and interspecific interactions upon community assembly. Velvet ants were sampled using 25 arrays of Y-shaped pitfall traps with drift fences for one year along an environmental gradient from cerrado *sensu stricto* (open canopy, warmer, drier) to cerradão (closed canopy, cooler, moister). Dataloggers installed on each trap recorded microclimate parameters throughout the study period. The effects of spatial distances, microclimate parameters and shared ancestry on species abundances and turnover were assessed with canonical correspondence analysis, generalized dissimilarity modelling and variance components analysis. Velvet ant diversity and abundance were higher in the cerrado *sensu stricto* and early in the wet season. There was pronounced compositional turnover along the environmental gradient, and temporal variation in richness and abundance was stronger than spatial variation. The dry season blooming of woody plant species fosters host abundance and, subsequently, velvet ant captures. Species were taxonomically clustered along the gradient with Sphaeropthalmina (especially *Traumatomutilla* spp.) and Pseudomethocina more associated, respectively, with cerrado *sensu stricto* and cerradão. This suggests a predominant role of environmental filters on community assemble, with physiological tolerances and host preferences being shared among members of the same lineages. Induced environmental changes in Cerrado can impact communities of wasps and their hosts with unpredictable consequences upon ecosystem functioning and services.

## Introduction

Ecological and historical factors interact in complex ways and operate at different scales during the assembly of biological communities [1–3]. At wide spatial-temporal scales, the role of speciation, extinction and dispersion is more prominent, whereas at local scales community assembly is mediated by neutral processes, environmental filters and ecological interactions [4–6]. Environmental filters promote the co-occurrence of species that share suitable traits, either due to inheritance from a common ancestor or due to convergence, whereas competition acts in the opposite direction, limiting the coexistence of similar species [7]; because related species tend to be more similar, environmental filters can result in phylogenetic clustering, whereas competition can result in phylogenetic overdispersion [3]. Yet, contemporary coexistence theory indicates that phylogenetic clustering can also result from large fitness differences and competitive exclusion, whereas phylogenetic overdispersion may arise from environmental filters acting on species with similar traits (when traits are convergent) or lack of stabilizing niche differences between closely related species (when traits are conserved) [8, 9]. The integration of phylogenies with ecological data obtained at different scales is regarded as essential in contemporary studies on community assembly [10–12]. However, this integration is limited by the Darwinian deficit, or the lack of knowledge on the phylogenetic relationships between species, especially in tropical regions [13]. Furthermore, the identification of relevant scales for conducting studies on community assembly is a major challenge [14, 15], especially in multitaxonomic approaches, given the great variation in body size, dispersal capacity and generation time between species [16–20].

Since the seminal works of Whittaker [21–23], ecological gradient analysis is used to understand the effects of spatial and environmental factors on species distributions and community assembly, as well as community boundaries and species turnover (beta diversity) at different spatial scales [24–26]. In addition, the effects of spatial distances and environmental gradients upon beta diversity are relevant to understanding the sensitivity of biological communities to changing environments, such as climate change [27–29] or invasive species [30–32]. Ecological gradient analysis has become popular with the development of community ordination methods [33] and their modern alternatives [34–36], as well as their implementation in a modular open source platform for statistical computing and graphics [37, 38].

In this work, we examined the roles of spatial distances, microclimate parameters, and taxonomic relationships upon the structure of velvet ant (Hymenoptera, Mutillidae) communities along a forest-savanna gradient in the Cerrado of central Brazil. Despite their common name, velvet ants are ectoparasitoid wasps that use larvae or pupae of other insects, mainly bees or other solitary wasps, as hosts [39, 40]. Therefore, they can regulate host populations and play a fundamental role in ecosystem functioning. Velvet ants comprise about 4200 species distributed mainly in the tropical region [41, 42]. They have marked sexual dimorphism, evidenced by the usually winged males as opposed to wingless females, but also reflected in body size, pattern of setae and coloration [43–46], which makes the taxonomy of the group complicated. The phylogenetic relationships among the major lineages of velvet ants are not well established [47, 48] and virtually nothing is known about the relationships among Neotropical species [49, 50]. Most Neotropical velvet ants are diurnal, more active early in the morning and late in the afternoon [40, 51], and seem to prefer open and dry environments [52–54]. Temperature plays an essential role in the development and survival of ectothermic parasitoids, as well as on their hosts and the asynchronous pattern of population fluctuations of both [55]. Therefore, temporal and spatial variation in temperature should be an important environmental filter affecting the abundance and composition of velvet ant communities.

Herein, the following questions were addressed: (1) does the abundance and richness of velvet ants vary predictably throughout the year and along the environmental gradient? (2) what are the relative roles of spatial distance and microclimate parameters on the community structure? and (3) are velvet ants taxonomically structured along the environmental gradient? By answering these questions, the goal was to understand the response of velvet ant communities to spatial and environmental variation, to assess the role of environmental filters and interspecific interactions on the assembly of velvet ant communities, and to discuss the potential impacts of environmental change upon these communities.

## Materials and methods

### Study area

**Fig 1:**
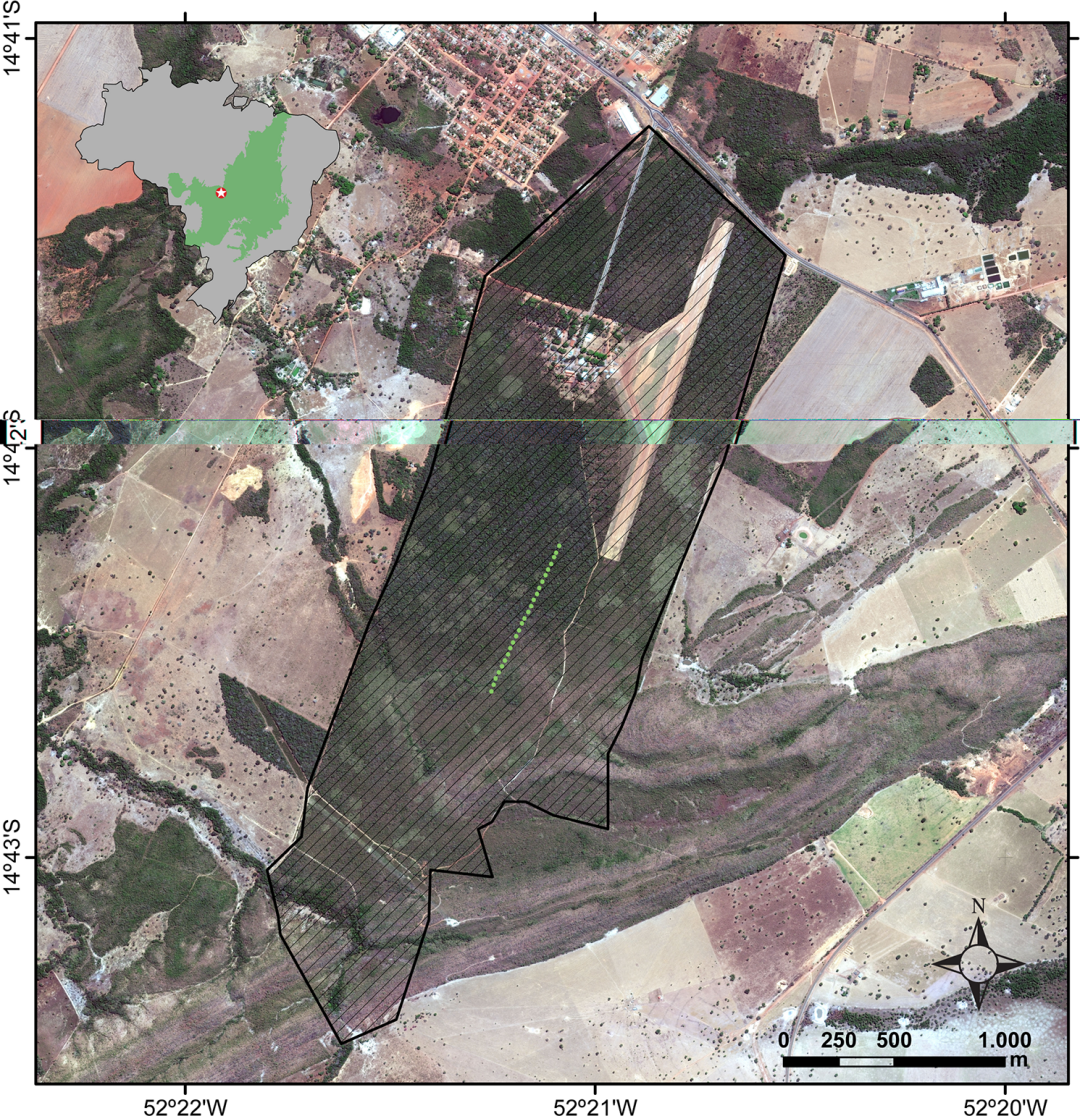
Study area. Parque Municipal Mário Viana, Nova Xavantina, Mato Grosso, Brazil. Within a transect (green points) and along an environmental gradient from cerrado *sensu stricto* (open canopy, drier, warmer) to cerradão (closed canopy, moister, cooler), 25 evenly spaced arrays of Y-shaped pitfall traps with drift fences were placed to sample velvet ants for 12 months; each array had a datalogger to record microclimate parameters of relative humidity and temperature. The overview map depicts the extent of Cerrado (light green) within Brazil (grey) and the municipality of Nova Xavantina location (white star).

This study was conducted at Parque Municipal Mário Viana, locally known as “Parque do Bacaba” (14°42’24” S, 52°21’9” W), in the municipality of Nova Xavantina, Mato Grosso state, Brazil (Fig 1). With 492 ha, it is located in the western portion of the Cerrado biome, close to its contact with Amazonia [56]. The park has forest habitats, such as “cerradão” (dense woodland savanna) or gallery forest, and savanna habitats, such as “cerrado rupestre” (saxicolous vegetation) or cerrado *sensu stricto* [57, 58]. The cerrado *sensu stricto* is characterized by trees 3–8 m high, abundant herb-grass stratum and a savannic appearance, whereas cerradão is characterized by trees 8–12 m high, closed canopy and a forest appearance [59–61]. The regional climate is markedly seasonal, with a dry season from May to September and a rainy season from October to April [62]. The annual precipitation varies between 1300 mm and 1500 mm and the mean annual temperature is 26°C [63].

### Sampling design

The data were collected in a forest-savanna gradient from cerrado *sensu stricto* (open canopy, warmer, drier) to cerradão (closed canopy, cooler, moister). Twenty-five arrays of pitfall traps with drift fences were installed at every 30 m along a linear transect of approximately 750 m. Each pitfall trap array consisted of four 35 l plastic buckets buried in the ground and arranged as a Y-shape, with the central bucket connected to three peripheral buckets by 6 m long and 50 cm high drift fences made of galvanized steel flat sheets. Traps, numbered 1 to 25 from cerrado *sensu stricto* to cerradão, were opened for one week every month from August 2015 to July 2016. When opened, traps were checked every day and velvet ants collected with forceps, stored in vials separated by traps, and preserved in 100% ethanol. Subsequently, velvet ants were mounted and identified (many to genus level or morphospecies, including several undescribed taxa). Only female velvet ants were represented in our samples. Fieldwork was conducted under permit SISBIO #335343 from Instituto Chico Mendes de Conservação da Biodiversidade (ICMBio).

### Microclimate parameters

Air temperature and relative humidity were recorded at each pitfall trap array, every 10 minutes during the entire study period with dataloggers (HOBO Pro v2 Temperature/ Relative Humidity, Onset Computer Corporation, MA, EUA). Dataloggers were installed two meters away from the central bucket, 50 cm high from the ground and sheltered from rain and direct solar radiation by a PVC hood.

Temperature and relative humidity records from dataloggers were averaged by hour/day/trap. Subsequently, monthly absolute values (minimum, maximum), means and standard deviations were estimated. This resulted in 14 microclimate parameters for each trap and month (25 traps X 12 months = 300 observations for each parameter): (1) absolute maximum relative humidity (*Hmaxa*), (2) absolute maximum temperature (*Tmaxa*), (3) absolute minimum relative humidity (*Hmina*), (4) absolute minimum temperature (*Tmina*), (5) absolute standard deviation of relative humidity (*Hsda*), (6) absolute standard deviation of temperature (*Tsda*), (7) maximum relative humidity (*Hmax*), (8) maximum temperature (*Tmax*), (9) mean relative humidity (*Hmean*), (10) mean temperature (*Tmean*), (11) minimum relative humidity (*Hmin*), (12) minimum temperature (*Tmin*), (13) standard deviation of relative humidity (*Hsd*), (14) standard deviation of temperature (*Tsd*). These parameters represent different microclimate aspects such as its central tendency and variability, as well as dominant monthly patterns and extreme values. A previous study showed that the microclimate is highly correlated with structural habitat and vegetation parameters, and is a good predictor of variation in the structure of velvet ant communities [52].

Due to technical problems with some dataloggers, microclimate parameters were not recorded in September 2015 in trap 25, in October 2015 in trap 25, in March 2016 in trap 6, and in July 2016 in trap 2 (4 in 300 or 1.33% missingness). Missing values can reduce the power of statistical analyses due to loss of information and degrees of freedom. Among the various methods available to estimate missing values [64, 65], multiple imputation is considered the most robust because it requires less assumptions about patterns of missing data [66, 67] and, in some cases, introduces less bias in analyses than the simple removal of missing data [68]. Missing values were estimated by averaging 100 imputations using package *Amelia II* [69], which employs an “expectation-maximization with bootstrapping” (EMB) algorithm suitable for time series data.

### Statistical analyses

To explore the associations among microclimate parameters, and between traps and microclimate parameters, a Principal Components Analysis (PCA) of the correlation matrix [70] was implemented with package *vegan* [71]. Species accumulation curves based on samples (pitfall trap arrays) and individuals were calculated to evaluate sampling sufficiency [72] with package *vegan*. The Chao1 index [73, 74] was used to estimate the asymptotic richness of the velvet ant community, while reducing the effects of subsampling and accounting for the presence of undetected species with package *vegan*. A Correspondence Analysis (CA) was used to explore the associations within species, and between species and traps, with package *CA* [75]. To reduce the influence of rare species, only those with number of captures higher than average were used [76]. Prior to analyses, species abundance data (number of captures) were transformed to log_10_. To represent the spatial-temporal variation in number of captures and richness, a surface graph was produced by fitting a local regression model using package *locfit* [77] with the default settings.

To identify the best environmental predictors of changes in community structure along the gradient, two approaches were used: Canonical Correspondence Analysis [78] and a combination of Generalized Dissimilarity Modelling [35] with variance partitioning [79]. The CCA was performed with package *vegan* and started with a null model, containing only the intersection, followed by the stepwise addition of microclimate parameters considering the Akaike Information Criterion (AIC). Model significance was determined by an analysis of variance (ANOVA) based on 1000 Monte Carlo randomizations. As in CA, only species with number of captures higher than average were used. Because GDM is not as widely used as PCA, CA or CCA in ecological studies, we provide more details about its principles and algorithm. GDM is a novel nonlinear matrix regression method that relates patterns of species turnover between pairs of sampling locations to environmental and geographic distances [80, 81]. While CCA associates raw species abundances with environmental (microclimate) parameters, GDM associates dissimilarity in species composition (e.g., beta diversity) to environmental dissimilarity and spatial distance between trap arrays. It has two major advantages over classical linear methods (like matrix regression, CCA), by accommodating (i) variation in the rate of species turnover and (ii) nonlinear relationships between species turnover and environmental/spatial separation. This is achieved (i) by nonlinear transformation of each environmental predictor using maximum likelihood and monotonic I-splines and (ii) by fitting GDM models using generalized linear modelling and specifying adequate link and variance functions [27, 35]. GDM was implemented with package *gdm* [82] using a Bray-Curtis dissimilarity matrix [83] to represent beta diversity, and the default settings.

To assess the existence of taxonomic structure in the distribution of velvet ants along the environmental gradient, a Variance Components Analysis (VCA) was used to describe the hierarchical effects of genera, subtribes, tribes and subfamilies (predictors) upon species preferences to microclimate (response). In the absence of information about phylogenetic relationships between velvet ant species, the taxonomic hierarchy was considered as its best approximation [84–86]. Species microclimate preferences were considered as their scores on the first axis of the CCA (above), which correspond approximately to the average of microclimate predictors weighted by species captures in each trap, i.e., their microclimate optima [78]. High components of variance indicate differences in microclimate preferences between taxonomic groups, and phylogenetic conservatism. The VCA was implemented with package *VCA* [87] through a linear mixed model (LME), where predictors were considered as random effects and model parameters were estimated through restricted maximum likelihood (REML) [88]. All statistical analyses were performed in R [38], using a 5% significance level.

## Results

**Fig 2:**
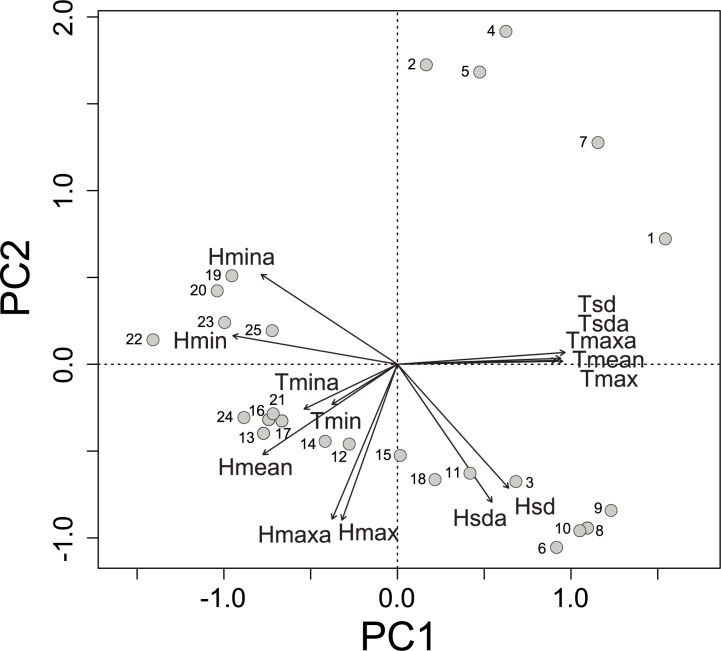
Environmental ordination. Principal Components Analysis (PCA) describing the variation of microclimatic parameters collected for 12 months in 25 arrays of Y-shaped pitfall traps with drift fences, along an environmental gradient from cerrado *sensu stricto* to cerradão at Parque Municipal Mário Viana, Nova Xavantina, Mato Grosso, Brazil. Filled circles represent pitfall trap arrays. Trap array numbers increase from cerrado *sensu stricto* to cerradão. The first two principal components (PC1 and PC2) explained 86% of the variation. Arrow length and direction are proportional to correlation between microclimatic parameters and ordination axes. Largest eigenvectors: PC1: *Tsda*, *Tsd*, *Hmin*, *Tmax*, *Tmaxa*, and *Tmean*; PC2: *Hmax*, *Hmaxa*, and *Hsda*. Legend: *Hmaxa*, absolute maximum relative humidity (%); *Hmina*, absolute minimum relative humidity (%); *Hmax*, maximum relative humidity (%); *Hmin*, minimum relative humidity (%); *Hmean*, mean relative humidity (%); *Hsda*, absolute standard deviation of relative humidity (%); *Hsd*, standard deviation of relative humidity (%); *Tmaxa*, absolute maximum temperature (°C); *Tmina*, absolute minimum temperature (°C); *Tmax*, maximum temperature (°C); *Tmin*, minimum temperature (°C); *Tmean*, mean temperature (°C); *Tsda*, absolute standard deviation of temperature (°C); *Tsd*, standard deviation of temperature (°C).

The first two PCA axes (Fig 2) jointly reduced 86.33% of the total variation in microclimatic parameters (PC1 = 60.39%, PC2 = 25.94%). This ordination described an environmental gradient of decreasing air temperature and increasing relative humidity from cerrado *sensu stricto* to cerradão (Fig 2, S1 Table).

**Fig 3:**
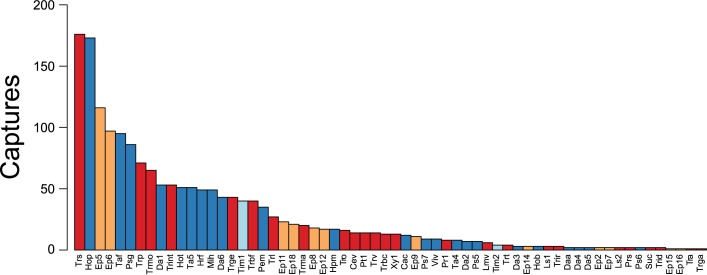
Relative abundance distribution. Bar plot of velvet ant species captured for 12 months in 25 arrays of Y-shaped pitfall traps with drift fences, along an environmental gradient from cerrado *sensu stricto* to cerradão at Parque Municipal Mário Viana, Nova Xavantina, Mato Grosso, Brazil. Bar colours code for the subtribe which the species pertain to; red: Sphaeropthalmina, blue: Pseudomethocina, orange: Ephutina, and light blue: Smicromyrmina. Legend: Cac, *Callomutilla crucigera;* Cev, *Cephalomutilla vivata;* Daa, *Darditilla araxa;* Da1, *Darditilla* sp. 01; Da2, *Darditilla* sp. 02; Da3, *Darditilla* sp. 03; Da4, *Darditilla* sp. 04; Da5, *Darditilla* sp. 05; Da6, *Darditilla* sp. 06; Ep2, *Ephuta* sp. 02; Ep5, *Ephuta* sp. 05; Ep6, *Ephuta* sp. 06; Ep7, *Ephuta* sp. 07; Ep8, *Ephuta* sp. 08; Ep9, *Ephuta* sp. 09; Ep11, *Ephuta* sp. 11; Ep12, *Ephuta* sp. 12; Ep14, *Ephuta* sp. 14; Ep15, *Ephuta* sp. 15; Ep16, *Ephuta* sp. 16; Ep18, *Ephuta* sp. 18; Hpm, *Hoplocrates monacha;* Hob, *Hoplomutilla biplagiata;* Hop, *Hoplomutilla pollens;* Hot, *Hoplomutilla triumphans;* Hrf, *Horcomutilla fronticornis;* Lmv, *Lophomutilla vina;* Ls1, *Lophostigma* sp. 01; Ls2, *Lophostigma* sp. 02; Mih, *Mickelia harpyia;* Pem, *Pertyella mayri;* Prs, *Protophotopsis sulcifrons;* Pr1, *Protophotopsis* sp. 01; Psg, *Pseudomethoca gounellei;* Ps5, *Pseudomethoca* sp. 05; Ps6, *Pseudomethoca* sp. 06; Ps7, *Pseudomethoca* sp. 07; Pt1, *Ptilomutilla* sp. 01; Suc, *Suareztilla centrolineata;* Taf, *Tallium festivum;* Ta4, *Tallium* sp. 04; Ta5, *Tallium* sp. 05; Tim1, *Timulla* sp. 01; Tim2, *Timulla* sp. 02; Tra, *Traumatomutilla andrei;* Trbc, *Traumatomutilla bellicosa;* Trbf, *Traumatomutilla bellifera;* Trga, *Traumatomutilla gausapata;* Trge, *Traumatomutilla geographica;* Trid, *Traumatomutilla indica;* Trint, *Traumatomutilla integella;* Trir, *Traumatomutilla ira;* Trl, *Traumatomutilla laida;* Trma, *Traumatomutilla maipa;* Trmo, *Traumatomutilla moesta;* Tro, *Traumatomutilla ocellaris;* Trp, *Traumatomutilla parallela;* Trs, *Traumatomutilla sancta;* Trt, *Traumatomutilla tristis;* Trv, *Traumatomutilla vivax;* Viv, *Vianatilla victura;* Xy1, *Xystromutilla* sp. 01.

**Fig 4:**
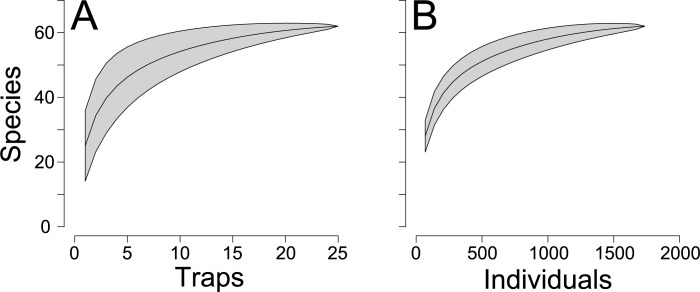
Rarefaction curves. Sample-based (A) and individual-based (B) rarefaction curves of velvet ants captured for 12 months in 25 arrays of Y-shaped pitfall traps with drift fences, along an environmental gradient from cerrado *sensu stricto* to cerradão at Parque Municipal Mário Viana, Nova Xavantina, Mato Grosso, Brazil.

**Fig 5:**
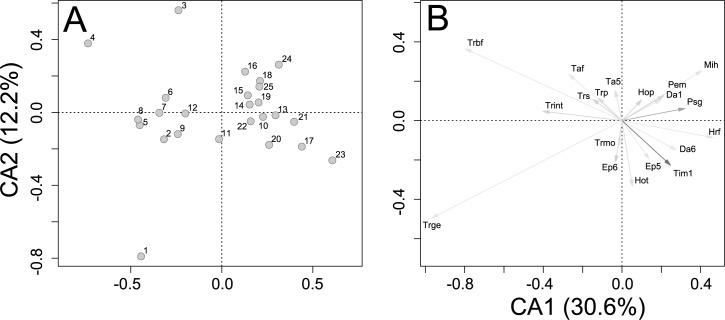
Species ordination. Correspondence analysis (CA) describing the variation on the distribution of velvet ants (only those species with abundance higher than average) captured for 12 months in 25 arrays of Y-shaped pitfall traps with drift fences, along an environmental gradient from cerrado *sensu stricto* to cerradão at Parque Municipal Mário Viana, Nova Xavantina, Mato Grosso, Brazil. Filled circles represent pitfall trap arrays. Trap array numbers increase from cerrado *sensu stricto* to cerradão. The first two correspondence axes (CA1 and CA2) explained 42.7% of the variation. Legend: Da1, *Darditilla* sp. 01; Da6, *Darditilla* sp. 06; Ep5, *Ephuta* sp. 05; Ep6, *Ephuta* sp. 06; Hop, *Hoplomutilla pollens*; Hot, *Hoplomutilla triumphans*; Hrf, *Horcomutilla fronticornis*; Mih, *Mickelia harpyia*; Pem, *Pertyella mayri*; Psg, *Pseudomethoca gounellei*; Taf, *Tallium festivum*; Ta5, *Tallium* sp. 05; Tim1, *Timulla* sp. 01; Trbf, *Traumatomutilla bellifera*; Trge, *Traumatomutilla geographica*; Trint, *Traumatomutilla integella*; Trmo, *Traumatomutilla moesta*; Trp, *Traumatomutilla parallela*; Trs, *Traumatomutilla sancta*.

**Fig 6:**
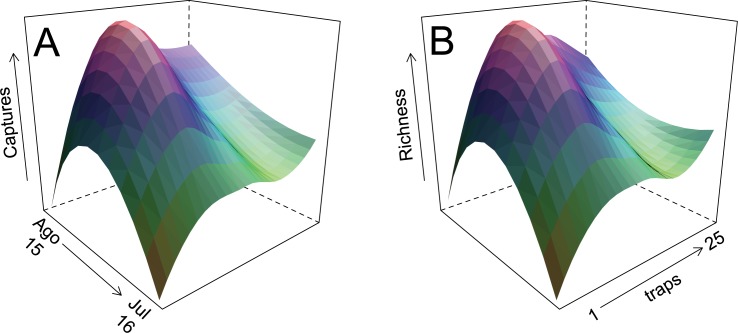
Captures and richness. Monthly distribution of velvet ant species captures and richness by traps, collected for 12 months in 25 arrays of Y-shaped pitfall traps with drift fences, along an environmental gradient from cerrado *sensu stricto* to cerradão at Parque Municipal Mário Viana, Nova Xavantina, Mato Grosso, Brazil. Trap array numbers increase from cerrado *sensu stricto* to cerradão.

Overall, 1737 individuals of velvet ant representing 62 species and 19 genera were collected during the study (Fig 3, S2 Table). The most abundant species were *Traumatomutilla sancta* Mickel, 1964 and *Hoplomutilla pollens* (Kohl, 1882). Species accumulation curves showed a tendency for stabilization, and few species would be added with increased sampling effort (Fig 4). The estimated richness (Chao1) of 63 ± 1.7 species resembled the observed richness, further indicating the quality of the sampling effort. The first two axes of the CA jointly reduced 42.8% of the total association between traps and velvet ant species (CA1 = 30.6%, CA2 = 12.2%). The ordination split traps in two groups, corresponding to cerrado *sensu stricto* and cerradão (Fig 5, S3 Table). Species of Sphaeropthalmina, mainly in the genus *Traumatomutilla* and especially *T*. *geographica* (Gerstaecker, 1874), *T*. *bellifera* (Cresson, 1902), and *T*. *integella* (Cresson, 1902), were strongly associated with traps (1–12) in cerrado *sensu stricto*, whereas species of Pseudomethocina, especially *Horcomutilla fronticornis* (Burmeister, 1854), *Mickelia harpyia* (Gerstaecker, 1874) and *Pseudomethoca gounellei* (André, 1895), were more associated with traps (13–25) in cerradão. Velvet ant richness and abundance were highest in the first half of the rainy season (November and December) and in the open portion of the gradient (cerrado *sensu stricto*); the temporal variation was more pronounced than the spatial variation (Fig 6).

**Fig 7:**
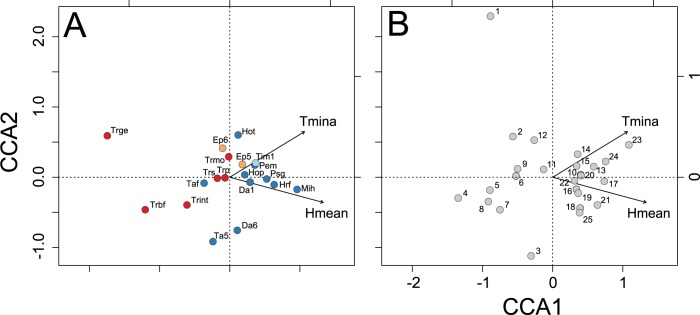
Constrained species ordination. Best (reduced) Canonical Correspondence Analysis (CCA) model, depicting relationships between (A) velvet ant species (with abundance higher than average) and microclimatic parameters, and (B) pitfall trap arrays and microclimatic parameters, collected for 12 months in 25 arrays of Y-shaped pitfall traps with drift fences, along an environmental gradient from cerrado *sensu stricto* to cerradão at Parque Municipal Mário Viana, Nova Xavantina, Mato Grosso, Brazil. Species scores are represented by abbreviations, microclimatic parameters by arrows and pitfall trap array position by circles and numbers. Coloured filled circles code for the subtribe which the species pertain to; red: Sphaeropthalmina, blue: Pseudomethocina, orange: Ephutina, and light blue: Smicromyrmina. Grey filled circles represent pitfall trap arrays. Trap array numbers increase from cerrado *sensu stricto* to cerradão. Jointly, graphs reflect species distributions along the microclimate parameter space. Arrow lengths represent strength of association between microclimate parameters and ordination axes. Legend: *Hmean*, mean relative humidity (%); *Tmina*, absolute minimum temperature (°C); Da1, *Darditilla* sp. 01; Da6, *Darditilla* sp. 06; Ep5, *Ephuta* sp. 05; Ep6, *Ephuta* sp. 06; Hop, *Hoplomutilla pollens*; Hot, *Hoplomutilla triumphans*; Hrf, *Horcomutilla fronticornis*; Mih, *Mickelia harpyia*; Pem, *Pertyella mayri*; Psg, *Pseudomethoca gounellei*; Taf, *Tallium festivum*; Ta5, *Tallium* sp. 05; Tim1, *Timulla* sp. 01; Trbf, *Traumatomutilla bellifera*; Trge, *Traumatomutilla geographica*; Trint, *Traumatomutilla integella*; Trmo, *Traumatomutilla moesta*; Trp, *Traumatomutilla parallela*; Trs, *Traumatomutilla sancta*.

The CCA model selection indicated mean relative humidity and absolute minimum temperature as the best microclimate predictors (*F*_2,22_ = 3.62, *P* < 0.05), reducing 23% of the variation in species abundances between traps. The CCA ordination of traps and species resembled the CA, revealing strong community structuring along the gradient (Fig 7, S4 Table). Species of Sphaeropthalmina, mostly in the genus *Traumatomutilla* and especially *T*. *geographica*, *T*. *bellifera* e *T*. *integella*, were strongly associated with traps characterized by lower mean relative humidity and lower absolute minimum temperature (cerrado *sensu stricto*, traps 1–12), whereas species of Pseudomethocina, especially *M*. *harpyia*, *H*. *fronticornis* and *P*. *gounellei*, exhibited an inverse association (cerradão, traps 13–25).

**Fig 8:**
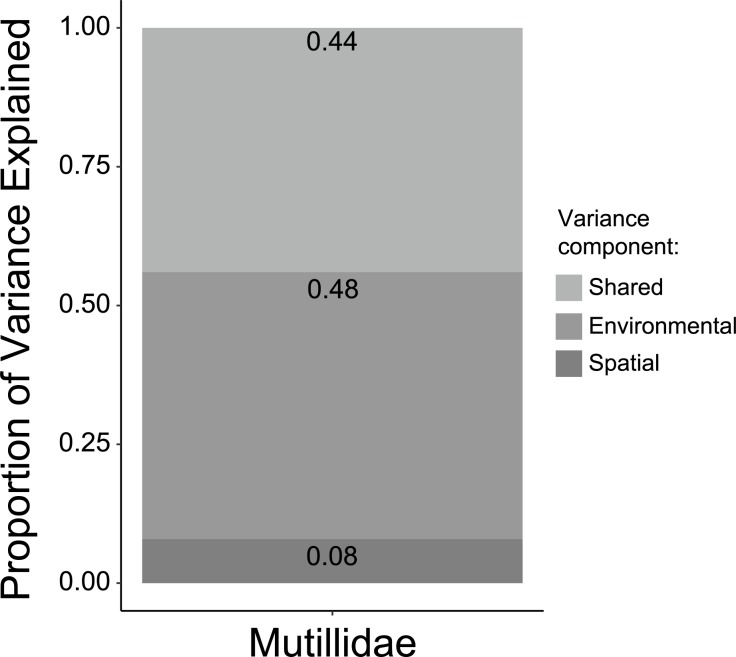
Variance partitioning. Partitioning of total deviance in velvet ant species turnover along an environmental gradient from cerrado *sensu stricto* to cerradão at Parque Municipal Mário Viana, Nova Xavantina, Mato Grosso, Brazil, according to Generalized Dissimilarity Modelling (GDM) using distance matrices of microclimate parameters and spatial distance as predictors. The stacked histogram represents fractions of total deviance in species turnover explained exclusively by each predictor distance matrix, their shared contribution, as well as deviance left unexplained.

**Fig 9:**
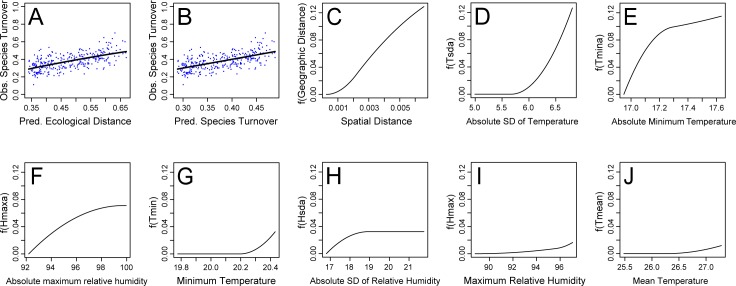
Species turnover. Generalized dissimilarity modelling of velvet ant species turnover across 25 arrays of Y-shaped pitfall traps with drift fences, distributed along an environmental gradient from cerrado *sensu stricto* to cerradão and sampled for 12 months in Nova Xavantina, Mato Grosso, Brazil. (A) Relationship between observed velvet ant turnover for each trap array pair and predicted ecological distance between those trap arrays. (B) Relationship between observed and predicted velvet ant turnover. Generalized dissimilarity model-fitted I-splines (partial regression fits) of spatial distance (C) and selected environmental variables (D-J) as predictors. The steeper the slope of the I-spline, the greater the predicted species turnover on that portion of the gradient. The maximum height of each curve indicates the total amount of species turnover associated with that predictor and the relative importance of that predictor in explaining species turnover holding all other predictors constant.

The Generalized Dissimilarity Modelling (GDM) indicated that microclimate parameters and spatial distances accounted for 36% of species turnover along the gradient. Spatial distances reduced only 8% of the explained variation (Fig 8) and GDM revealed species turnover across all distances encompassed by the transect (Fig 9A–9C). High covariance was detected between beta diversity explained by microclimatic parameters and spatial distances, corresponding to 44% of the explained variation (Fig 8). Environmental conditions reduced most (48%) of the explained variation in species turnover (Fig 8), the best predictors being absolute minimum temperature, mean temperature, absolute maximum relative humidity and standard deviation of temperature (S5 Table). Species turnover was pronounced at low values of absolute minimum temperature and decreased at intermediate values (Fig 9E). In contrast, there was gradual species turnover for low and intermediate values of standard deviation of temperature, but turnover increased rapidly above this limit (Fig 9D). A continuous species turnover was observed with increasing values of absolute maximum relative humidity (Fig 9F).

**Fig 10:**
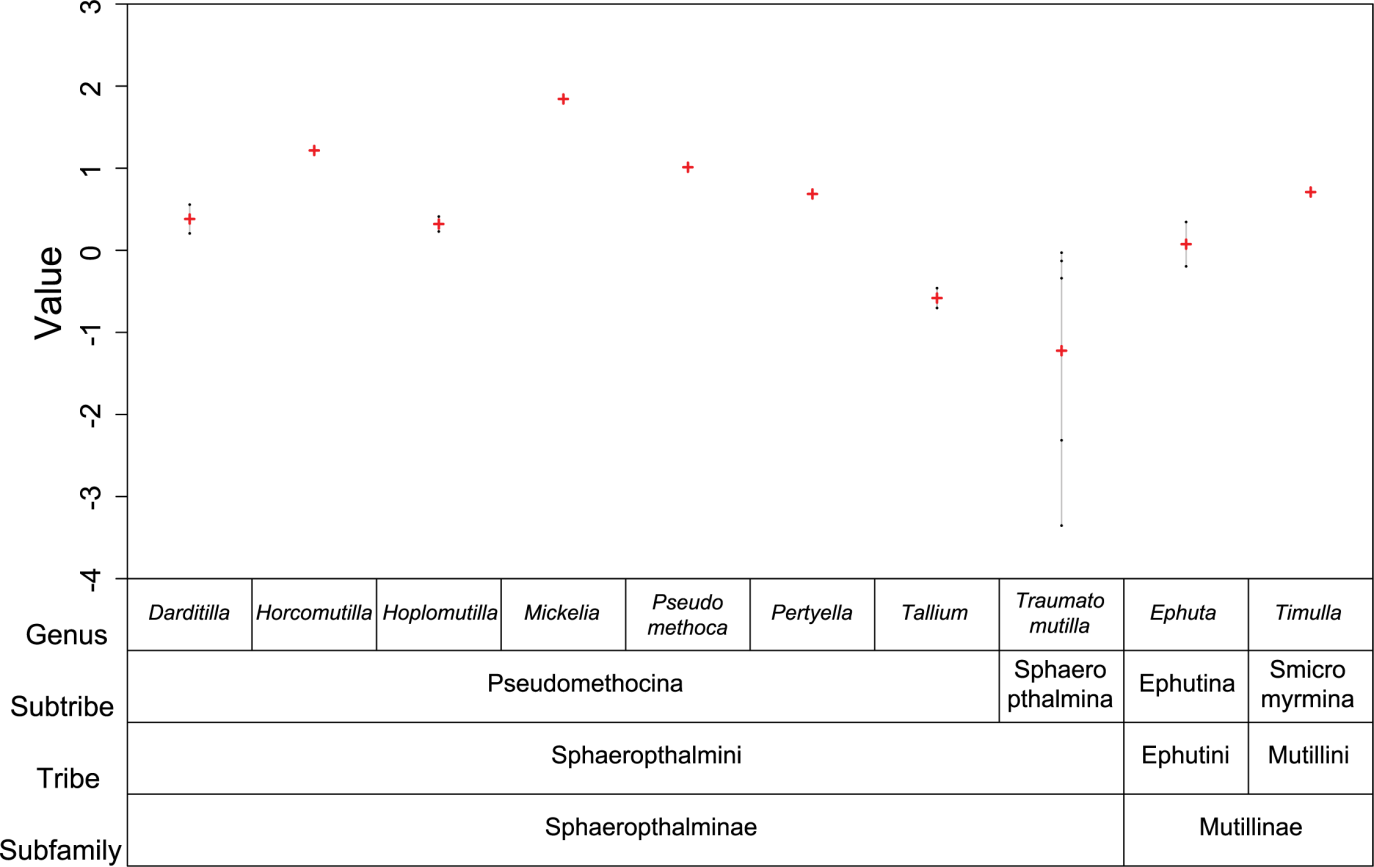
Variance Components Analysis. Variance Components Analysis (VCA) of microclimate preferences among taxonomic levels of velvet ants captured for 12 months in 25 arrays of Y-shaped pitfall traps with drift fences, along an environmental gradient from cerrado *sensu stricto* to cerradão at Parque Municipal Mário Viana, Nova Xavantina, Mato Grosso, Brazil. VCA used Restricted Maximum Likelihood (REML) to fit a linear mixed model (LMM) where the intercept is the only fixed effect. Values represent means of species scores along the first axis of a Canonical Correspondence Analysis relating number of captures to microclimate parameters and are distributed among four taxonomic levels (subfamily, tribe, subtribe and genus).

The VCA indicated significant taxonomic structure in microclimate preferences above the species level, with virtually all variation concentrated among subtribes (Table 1). Species of Sphaeropthalmina, all in the genus *Traumatomutilla*, showed preference for portions of the gradient with lower values of mean relative humidity and absolute minimum temperature when compared to species in other subtribes (Fig 10).

**Table 1:** Variance Components Analysis.

Source of variation	df	VC	%Total	SD	CV[%]	Var(VC)
Total	7.54	1.56	100	1.25	-1597.82	0.64
Subfamily	—	0.00	0.00	0.00	0.00	0.00
Subfamily:Tribe	—	0.00	0.00	0.00	0.00	0.00
Subfamily:Tribe:Subtribe	1.28	0.62	39.87	0.79	-1008.94	0.60
Subfamily:Tribe:Subtribe:Genus	—	0.00	0.00	0.00	0.00	0.00
Species (Error)	15.17	0.94	60.13	0.97	-1238.98	0.12

Variance Components Analysis (VCA) of microclimate preferences among taxonomic levels of velvet ants captured for 12 months in 25 arrays of Y-shaped pitfall traps with drift fences, along an environmental gradient from cerrado *sensu stricto* to cerradão at Parque Municipal Mário Viana, Nova Xavantina, Mato Grosso, Brazil. VCA used Restricted Maximum Likelihood (REML) to fit a linear mixed model (LMM) where the intercept is the only fixed effect. Values are distributed among four taxonomic levels (subfamily, tribe, subtribe and genus). df: degrees of freedom, VC: variance component, SD: standard deviation, CV: coefficient of variation, Var(VC): variance of VC estimates.

## Discussion

Changes in vegetation structure along the environmental gradient from cerrado *sensu stricto* to cerradão gradient promoted important microclimate shifts at the ground level. Open areas are more exposed to higher solar radiation, being hotter and drier, and experiencing greater variation in temperature and humidity. Besides the spatial variation, there is a pronounced seasonality in the Cerrado, characterized by a regular and predictable variation in precipitation along the year. This spatial-temporal variation in environmental conditions is reflected in the structure of velvet ant communities, where species abundance and richness peak in the warmer and drier portions of the gradient and in the first half of the rainy season, as well as in the patterns of species turnover along the gradient. The different combinations of microclimate parameters can define favourable conditions in small spatial-temporal scales and promote the coexistence of a greater number of species when compared to more constant environments [89]. To a certain extent, this may explain the high species richness recorded in this study compared to other studies conducted in the Neotropical region [52, 90–93]. Yet, these differences can be attributed to variation in sampling methods and effort. For instance, pitfall traps with drift fences seem much more efficient than Malaise or Moericke traps in capturing velvet ants [52, 90, 94]. Further, the proximity of the study area to the Amazonia–Cerrado transition can provide conditions and resources for species from both biomes [52, 95, 96]. Ecotones may exhibit greater biodiversity than adjacent areas due to additive processes and the presence of endemic species [97, 98], playing an important role in lineage diversification and biodiversity conservation [99, 100].

Peaks of abundance in the first half of the rainy season seem to be a pattern among velvet ants in the Neotropical region [90, 94]. Because velvet ants are ectoparasitoids of larvae or pupae, mainly of other Aculeata [101–104], they should show abundance peaks coincident with the end of the provisioning period of their hosts in every generation, which results in asynchronous abundance peaks between parasitoids and their hosts [40, 105, 106]. Accurate data on hosts of Cerrado velvet ants are wanting; however, most Cerrado woody plants are primarily pollinated by animals and bloom at the end of the dry season [107–111], providing plenty of food resources for host populations, which also peak at the same period [90, 101, 112, 113]. Therefore, the peak abundance of velvet ants during the first half of the rainy season occurs shortly after the abundance peaks of their hosts. Yet, because velvet ants are ectotherms, the low temperatures during the dry months can promote a reduction in their activity [114, 115].

The open portion of the environmental gradient (cerrado *sensu stricto*) concentrated greater richness and abundance of velvet ants. Like the temporal variation, this could stem from species physiological tolerances to temperature and humidity close to the soil surface, as well as the distribution of host species along the gradient. The greater environmental heterogeneity and the milder microclimate of forest areas provide greater diversity of bees and wasps [116–119]; nevertheless, velvet ants seem to be more abundant and diverse overall in open areas [52–54]. In the Neotropics, this seems to be associated with preference for open areas of two potential host groups, Crabronidae and Sphecidae [90, 102, 120]. Further, parasitoid abundance and rates of parasitism are often higher in open environments or in edges resultant from habitat fragmentation [121, 122]. Also, velvet ants may be better adapted to tolerate variations in temperature and relative humidity, i.e., environments with less vegetation and more exposed soil are suitable for the establishment of these organisms [53]. The strong taxonomic structuring of velvet ants along the gradient indicates a predominant role of environmental filters in community organization. Apparently, physiological tolerances to microclimatic parameters or preferences for certain host species, which determine species turnover along the gradient, are inherited and shared by members of the same lineages (subtribes).

The Cerrado, a global biodiversity hotspot, largest and most diverse tropical savanna, sustains accelerated loss of natural habitats due to the expansion of agricultural activities and habitat remnants undergo periodic fires and are subject to edge effects [123–125]. Moreover, the biota has been affected by local climate change, characterized by reduced rainfall and increased temperature [126–128]. All these factors should contribute to significant changes in the structure of velvet ant communities, especially favouring those lineages more adapted to open habitats (Sphaeropthalmina), to the detriment of the lineages most associated to areas with denser vegetation cover (Ephutini, Pseudomethocina and Smicromyrmina). These changes, in turn, will certainly affect host populations with unpredictable consequences to the trophic cascade and the environmental services they provide.

## Supporting information

S1 Table Principal Components Analysis. Principal Components Analysis (PCA) of microclimate parameters recorded for 12 months in 25 arrays of Y-shaped pitfall traps with drift fences, along an environmental gradient from cerrado *sensu stricto* to cerradão at Parque Municipal Mário Viana, Nova Xavantina, Mato Grosso, Brazil.
(DOCX)

S2 Table Species of velvet ants. **S**pecies of velvet ants captured for 12 months in 25 arrays of Y-shaped pitfall traps with drift fences, along an environmental gradient from cerrado *sensu stricto* to cerradão at Parque Municipal Mário Viana, Nova Xavantina, Mato Grosso, Brazil.
(DOCX)

S3 Table Correspondence Analysis. Correspondence Analysis (CA) of velvet ant captures for 12 months in 25 arrays of Y-shaped pitfall traps with drift fences, along an environmental gradient from cerrado *sensu stricto* to cerradão at Parque Municipal Mário Viana, Nova Xavantina, Mato Grosso, Brazil.
(DOCX)

S4 Table Canonical Correspondence Analysis. Canonical Correspondence Analysis (CCA) of microclimate predictors and velvet ants captured for 12 months in 25 arrays of Y-shaped pitfall traps with drift fences, along an environmental gradient from cerrado *sensu stricto* to cerradão at Parque Municipal Mário Viana, Nova Xavantina, Mato Grosso, Brazil.
(DOCX)

S5 Table Generalized Dissimilarity Modelling. Generalized Dissimilarity Modelling (GDM) of spatial and microclimate predictors of turnover in velvet ants captured for 12 months in 25 arrays of Y-shaped pitfall traps with drift fences, along an environmental gradient from cerrado *sensu stricto* to cerradão at Parque Municipal Mário Viana, Nova Xavantina, Mato Grosso, Brazil. Values depict coefficients of three I-spline basis functions that represent the amount of compositional turnover associated with each predictor. The sum of the three coefficients represents the importance of each predictor in determining patterns of beta diversity.
(DOCX)
