# Supplementary material for: Spatial-temporal dynamics of neotropical velvet ant (Hymenoptera: Mutillidae) communities along a forest-savanna gradient
Source: PLoS One. 2017 Oct 27;12(10):e0187142. doi: 10.1371/journal.pone.0187142 (PMC5659792; doi:10.1371/journal.pone.0187142)
Supplement: S1 Table — Principal Components Analysis (PCA) of microclimate parameters recorded for 12 months in 25 arrays of Y-shaped pitfall traps with drift fences, along an environmental gradient from cerrado sensu stricto to cerradão at Parque Municipal Mário Viana, Nova Xavantina, Mato Grosso, Brazil. (DOCX) [file pone.0187142.s001.docx]

**Table S1. Principal Components Analysis.** Principal Components Analysis (PCA) of microclimate parameters recorded during 12 months with 25 Y-shaped pitfall traps with drift fences, along a cerrado *sensu stricto* – cerradão environmental gradient at Parque Municipal Mário Viana, Nova Xavantina, Mato Grosso, Brazil.

|  | Eigenvectors | |
| --- | --- | --- |
| Predictors | **PC1** | **PC2** |
| Absolute minimum temperature (*Tmina*) | -0.63 | -0.30 |
| Absolute minimum relative humidity (*Hmina*) | -0.92 | 0.61 |
| Absolute maximum temperature (*Tmaxa*) | 1.11 | 0.04 |
| Absolute maximum relative humidity (*Hmaxa*) | -0.44 | -1.05 |
| Absolute standard deviation of temperature (*Tsda*) | 1.14 | 0.08 |
| Absolute standard deviation of relative humidity (*Hsda*) | 0.64 | -0.93 |
| Mean temperature (*Tmean*) | 1.08 | 0.03 |
| Mean relative humidity (*Hmean*) | -0.91 | -0.61 |
| Minimum temperature (*Tmin*) | -0.44 | -0.27 |
| Minimum relative humidity (*Hmin*) | -1.12 | 0.19 |
| Maximum temperature (*Tmax*) | 1.12 | 0.02 |
| Maximum relative humidity (*Hmax*) | -0.38 | -1.06 |
| Standard deviation of temperature (*Tsd*) | 1.14 | 0.08 |
| Standard deviation of relative humidity (*Hsd*) | 0.75 | -0.84 |
| *Eigenvalue* | 8.45 | 3.63 |
| Proportion explained (%) | 60.0 | 26.0 |
| Cumulative proportion (%) | 60.0 | 86.0 |
