# Supplementary material for: Spatial-temporal dynamics of neotropical velvet ant (Hymenoptera: Mutillidae) communities along a forest-savanna gradient
Source: PLoS One. 2017 Oct 27;12(10):e0187142. doi: 10.1371/journal.pone.0187142 (PMC5659792; doi:10.1371/journal.pone.0187142)
Supplement: S2 Table — Species of velvet ants captured for 12 months in 25 arrays of Y-shaped pitfall traps with drift fences, along an environmental gradient from cerrado sensu stricto to cerradão at Parque Municipal Mário Viana, Nova Xavantina, Mato Grosso, Brazil. (DOCX) [file pone.0187142.s002.docx]

**Table S2. Species of velvet ants. S**pecies of velvet ants captured during 12 months with 25 Y-shaped pitfall traps with drift fences, along a cerrado *sensu stricto* – cerradão environmental gradient at Parque Municipal Mário Viana, Nova Xavantina, Mato Grosso, Brazil.

| Species |
| --- |
| Subfamily Mutillinae |
| Tribe Ephutini |
| Subtribe Ephutina |
| *Ephuta* sp. 02 |
| *Ephuta* sp. 05 |
| *Ephuta* sp. 06 |
| *Ephuta* sp. 07 |
| *Ephuta* sp. 08 |
| *Ephuta* sp. 09 |
| *Ephuta* sp. 11 |
| *Ephuta* sp. 12 |
| *Ephuta* sp. 14 |
| *Ephuta* sp. 15 |
| *Ephuta* sp. 16 |
| *Ephuta* sp. 18 |
| Tribe Mutillini |
| Subtribe Smicromyrmina |
| *Timulla* sp. 01 |
| *Timulla* sp. 02 |
| Subfamily Sphaeropthalminae |
| Tribe Sphaeropthalmini |
| Subtribe Pseudomethocina |
| *Callomutilla crucigera* (Burmeister, 1854) |
| *Darditilla araxa* (Cresson, 1902) |
| *Darditilla* sp. 01 |
| *Darditilla* sp. 02 |
| *Darditilla* sp. 03 |
| *Darditilla* sp. 04 |
| *Darditilla* sp. 05 |
| *Darditilla* sp. 06 |
| *Hoplocrates monacha* (Gerstaecker, 1874) |
| *Hoplomutilla biplagiata* Mickel, 1939 |
| *Hoplomutilla pollens* (Kohl, 1882) |
| *Hoplomutilla triumphans* Mickel, 1939 |
| *Horcomutilla fronticornis* (Burmeister, 1854) |
| *Mickelia harpyia* (Gerstaecker, 1874) |
| *Pertyella mayri* |
| *Pseudomethoca gounellei* (André, 1906) |
| *Pseudomethoca* sp. 05 |
| *Pseudomethoca* sp. 06 |
| *Pseudomethoca* sp. 07 |
| *Tallium festivum* (Smith, 1855) |
| *Tallium* sp. 04 |
| *Tallium* sp. 05 |
| *Vianatilla victura* (Cresson, 1902) |
| Subtribe Sphaeropthalmina |
| *Cephalomutilla vivata* (Cresson, 1902) |
| *Lophomutilla vina* Fritz & Pagliano, 1993 |
| *Lophostigma* sp. 01 |
| *Lophostigma* sp. 02 |
| *Protophotopsis* sp. 01 |
| *Protophotopsis sulcifrons* |
| *Ptilomutilla* sp. 01 |
| *Suareztilla centrolineata* (André, 1906) |
| *Traumatomutilla andrei* (Cresson, 1902) |
| *Traumatomutilla bellicosa* (Cresson, 1902) |
| *Traumatomutilla bellifera* (Cresson, 1902) |
| *Traumatomutilla gausapata* Mickel, 1952 |
| *Traumatomutilla geographica* (Gerstaecker, 1874) |
| *Traumatomutilla indica* Mickel, 1952 |
| *Traumatomutilla integella* (Cresson, 1902) |
| *Traumatomutilla ira* Casal, 1969 |
| *Traumatomutilla laida* Casal, 1969 |
| *Traumatomutilla maipa* Casal, 1969 |
| *Traumatomutilla moesta* Mickel, 1964 |
| *Traumatomutilla ocellaris* (Klug, 1821) |
| *Traumatomutilla parallela* Mickel, 1964 |
| *Traumatomutilla sancta* Mickel, 1964 |
| *Traumatomutilla tristis* |
| *Traumatomutilla vivax* (Gerstaecker, 1854) |
| *Xystromutilla* sp. 01 |
