# Supplementary material for: Spatial-temporal dynamics of neotropical velvet ant (Hymenoptera: Mutillidae) communities along a forest-savanna gradient
Source: PLoS One. 2017 Oct 27;12(10):e0187142. doi: 10.1371/journal.pone.0187142 (PMC5659792; doi:10.1371/journal.pone.0187142)
Supplement: S3 Table — Correspondence Analysis (CA) of velvet ant captures for 12 months in 25 arrays of Y-shaped pitfall traps with drift fences, along an environmental gradient from cerrado sensu stricto to cerradão at Parque Municipal Mário Viana, Nova Xavantina, Mato Grosso, Brazil. (DOCX) [file pone.0187142.s003.docx]

**Table S3. Correspondence Analysis.** Correspondence Analysis (CA) of velvet ant captures during 12 months with 25 Y-shaped pitfall traps with drift fences, along a cerrado *sensu stricto* – cerradão environmental gradient at Parque Municipal Mário Viana, Nova Xavantina, Mato Grosso, Brazil.

|  | Eigenvectors | |
| --- | --- | --- |
| Species | **CA1** | **CA2** |
| *Darditilla* sp. 06 | 0.88 | -0.76 |
| *Darditilla* sp. 01 | 0.63 | 0.54 |
| *Ephuta* sp. 05 | 0.44 | -0.99 |
| *Ephuta* sp. 06 | -0.10 | -1.06 |
| *Horcomutilla fronticornis* | 1.48 | -0.44 |
| *Hoplomutilla pollens* | 0.32 | 0.53 |
| *Hoplomutilla triumphans* | 0.17 | -1.69 |
| *Mickelia harpyia* | 1.30 | 1.28 |
| *Pseudomethoca gounellei* | 1.02 | 0.31 |
| *Pertyella mayri* | 0.68 | 0.65 |
| *Timulla* sp. 01 | 0.79 | -1.15 |
| *Tallium* sp. 05 | -0.11 | 0.78 |
| *Traumatomutilla bellifera* | -2.54 | 1.85 |
| *Tallium festivum* | -0.86 | 1.19 |
| *Traumatomutilla geographica* | -3.08 | -2.50 |
| *Traumatomutilla integella* | -1.28 | 0.24 |
| *Traumatomutilla moesta* | -0.05 | -0.65 |
| *Traumatomutilla parallela* | -0.48 | 0.55 |
| *Traumatomutilla sancta* | -0.39 | 0.58 |
| Eigenvalue | 0.10 | 0.04 |
| Proportion explained (%) | 30.6 | 12.2 |
| Cumulative proportion (%) | 30.6 | 42.7 |
