# Supplementary material for: Spatial-temporal dynamics of neotropical velvet ant (Hymenoptera: Mutillidae) communities along a forest-savanna gradient
Source: PLoS One. 2017 Oct 27;12(10):e0187142. doi: 10.1371/journal.pone.0187142 (PMC5659792; doi:10.1371/journal.pone.0187142)
Supplement: S4 Table — Canonical Correspondence Analysis (CCA) of microclimate predictors and velvet ants captured for 12 months in 25 arrays of Y-shaped pitfall traps with drift fences, along an environmental gradient from cerrado sensu stricto to cerradão at Parque Municipal Mário Viana, Nova Xavantina, Mato Grosso, Brazil. (DOCX) [file pone.0187142.s004.docx]

**Table S4. Canonical Correspondence Analysis.** Canonical Correspondence Analysis (CCA) of microclimate predictors and velvet ant captures during 12 months with 25 Y-shaped pitfall traps with drift fences, along a cerrado *sensu stricto* – cerradão environmental gradient at Parque Municipal Mário Viana, Nova Xavantina, Mato Grosso, Brazil.

|  | Eigenvectors | |
| --- | --- | --- |
| Predictors | **CCA1** | **CCA2** |
| Absolute minimum temperature (*Tmina*) | 0.74 | 0.67 |
| Mean relative humidity (*Hmean*) | 0.93 | -0.37 |
| Species |  |  |
| *Darditilla* sp. 06 | 0.06 | -0.27 |
| *Darditilla* sp. 01 | 0.15 | -0.02 |
| *Ephuta* sp. 05 | 0.09 | 0.06 |
| *Ephuta* sp. 06 | -0.05 | 0.15 |
| *Horcomutilla fronticornis* | 0.33 | -0.04 |
| *Hoplomutilla pollens* | 0.11 | 0.01 |
| *Hoplomutilla triumphans* | 0.06 | 0.21 |
| *Mickelia harpyia* | 0.50 | -0.06 |
| *Pseudomethoca gounellei* | 0.27 | -0.01 |
| *Pertyella mayri* | 0.19 | 0.06 |
| *Timulla* sp. 01 | 0.19 | 0.07 |
| *Tallium* sp. 05 | -0.12 | -0.32 |
| *Traumatomutilla bellifera* | -0.63 | -0.16 |
| *Tallium festivum* | -0.19 | -0.03 |
| *Traumatomutilla geographica* | -0.91 | 0.21 |
| *Traumatomutilla integella* | -0.32 | -0.14 |
| *Traumatomutilla moesta* | -0.01 | 0.10 |
| *Traumatomutilla parallela* | -0.04 | 0.00 |
| *Traumatomutilla sancta* | -0.09 | -0.01 |
| Eigenvalue | 0.07 | 0.02 |
| Proportion explained (total) | 0.23 | 0.05 |
| Cumulative proportion (total) | 0.23 | 0.28 |
| Proportion explained (constrained) | 0.83 | 0.17 |
| Cumulative proportion (constrained) | 0.83 | 1.00 |
