# Supplementary material for: Spatial-temporal dynamics of neotropical velvet ant (Hymenoptera: Mutillidae) communities along a forest-savanna gradient
Source: PLoS One. 2017 Oct 27;12(10):e0187142. doi: 10.1371/journal.pone.0187142 (PMC5659792; doi:10.1371/journal.pone.0187142)
Supplement: S5 Table — Generalized Dissimilarity Modelling (GDM) of spatial and microclimate predictors of turnover in velvet ants captured for 12 months in 25 arrays of Y-shaped pitfall traps with drift fences, along an environmental gradient from cerrado sensu stricto to cerradão at Parque Municipal Mário Viana, Nova Xavantina, Mato Grosso, Brazil. Values depict coefficients of three I-spline basis functions that represent the amount of compositional turnover associated with each predictor. The sum of the three coefficients represents the importance of each predictor in determining patterns of beta diversity. (DOCX) [file pone.0187142.s005.docx]

**Table S5. Generalized Dissimilarity Modelling.** Generalized Dissimilarity Modelling (GDM) of spatial and microclimate predictors of turnover in velvet ants captured during 12 months with 25 Y-shaped pitfall traps with drift fences, along a cerrado *sensu stricto* – cerradão environmental gradient at Parque Municipal Mário Viana, Nova Xavantina, Mato Grosso, Brazil. Values depict coefficients of three I-spline basis functions that represent the amount of compositional turnover associated with each predictor. The sum of the three coefficients represents the importance of each predictor in determining patterns of beta diversity.

| Predictor | Coef 1 | Coef 2 | Coef 3 | Sum |
| --- | --- | --- | --- | --- |
| Geographic distance | 0.000 | 0.095 | 0.035 | 0.129 |
| Absolute standard deviation of temperature (*Tsda*) | 0.000 | 0.000 | 0.129 | 0.129 |
| Absolute minimum temperature (*Tmina*) | 0.092 | 0.013 | 0.010 | 0.115 |
| Absolute maximum relative humidity (*Hmaxa*) | 0.071 | 0.000 | 0.000 | 0.071 |
| Minimum temperature (*Tmin*) | 0.000 | 0.000 | 0.034 | 0.034 |
| Absolute standard deviation of relative humidity (*Hsda*) | 0.032 | 0.000 | 0.000 | 0.032 |
| Maximum relative humidity (*Hmax*) | 0.000 | 0.009 | 0.008 | 0.017 |
| Mean temperature (*Tmean*) | 0.000 | 0.000 | 0.012 | 0.012 |
| Standard deviation of temperature (*Tsd*) | 0.000 | 0.000 | 0.000 | 0.000 |
| Absolute minimum relative humidity (*Hmina*) | 0.000 | 0.000 | 0.000 | 0.000 |
| Absolute maximum temperature (*Tmaxa*) | 0.000 | 0.000 | 0.000 | 0.000 |
| Mean relative humidity (*Hmean*) | 0.000 | 0.000 | 0.000 | 0.000 |
| Minimum relative humidity (*Hmin*) | 0.000 | 0.000 | 0.000 | 0.000 |
| Maximum temperature (*Tmax*) | 0.000 | 0.000 | 0.000 | 0.000 |
| Standard deviation of relative humidity (*Hsd*) | 0.000 | 0.000 | 0.000 | 0.000 |
